# Use of Chinese medicine correlates negatively with the consumption of conventional medicine and medical cost in patients with uterine fibroids: a population-based retrospective cohort study in Taiwan

**DOI:** 10.1186/s12906-015-0645-0

**Published:** 2015-04-23

**Authors:** Shan-Yu Su, Chih-Hsin Muo, Donald E Morisky

**Affiliations:** Department of Chinese Medicine, China Medical University Hospital, No. 2 Yude road, Taichung, 40447 Taiwan; School of Post-baccalaureate Chinese Medicine, College of Chinese Medicine, China Medical University, Taichung, 40402 Taiwan; College of Medicine, China Medical University, Taichung, 40402 Taiwan; Management Office for Health Data, China Medical University Hospital, Taichung, 40402 Taiwan; Department of Community Health Sciences, UCLA Fielding School of Public Health, Los Angeles, CA 90095-1772 USA

**Keywords:** Uterine fibroid, Chinese medicine, Conventional western medicine, Iron-deficiency anemia, Hypermenorrhea, Dysmenorrhea

## Abstract

**Background:**

Chinese medicine is commonly used and covered by health insurance to treat symptoms of uterine fibroids in Taiwan. This retrospective cohort study compared the consumption of conventional western medicine and medical cost between Chinese medicine (CM) users and nonusers among patients with uterine fibroids.

**Methods:**

We extracted 44,122 patients diagnosed with uterine fibrosis between 1996 and 2010 from the National Health Insurance reimbursement database, which is a population-based database released by a government-run health insurance system. Multivariate linear regression models were used to find association between using Chinese medicine and the consumption of conventional medicine, and between using Chinese medicine and medical cost.

**Results:**

The total fibroid-related conventional western medicine consumed by CM users was less than that by nonusers (β = -10.49, *P* < 0.0001). Three categories of conventional medicines, including antianemics (-3.50 days/year/patient, *P* < 0.0001), hemostatics (- 1.89 days/year/patient, *P* < 0.0001), and hormone-related agents (-3.13 days/year/patient, *P* < 0.0001), were used less in patients who were CM users. Moreover, although using CM increased 16.9 USD per patient in CM users annually (*P* < 0.0001), the total annual medical cost for treating fibroid was 5610 USD less in CM users than in nonusers (*P* < 0.0001).

**Conclusions:**

Our results suggested that CM reduced the consumption of conventional medicine, and might be a potential therapeutic substitute for conventional western medicines to treat uterine fibroids with low cost.

## Background

Uterine fibroids are the most common benign uterine tumors, which happens in 60% of reproductive aged women [1]. The majority of fibroids are asymptomatic, while in 20% of patients it causes symptoms such as hypermenorrhea, dysmenorrhea, iron-deficiency anemia, and infertility [2]. In symptomatic patients, the first-line treatment is surgery. However, since surgery often cause complications, conservative medical treatment to control symptoms related to fibroids are frequently adopted by patients and gynecologists [3]. Available indicated medical treatments include antianemics (blood derivatives and iron preparations) to treat iron-deficient anemia, hemostatics (coagulants and anti-fibrinolytic agents) to reduce bleedings of hypermenorrhea, and non-steroid anti-inflammatory drugs (NSAIDs) to relief pain. As no currently validated medical treatment is capable of making fibroid disappear, there are no reason to consider medical treatment in case of asymptomatic fibroids [4].

The estimated direct cost related to uterine fibroids includes cost of surgery, hospital admission, outpatient visit, and medication. The annual cost of uterine fibroids is up to 4.1-9.4 billion US dollars (USD) in the United States [5,6], including 3.27 to 5.10 billion USD in nonsurgical management and 829 million to 4.3 billion USD annually in surgical management [5]. If the indirect cost, including lost work-hour costs and obstetric outcomes cost, is taken into account, uterine fibroids are estimated to cost the United States 5.9 - 34.4 billion USD annually [5]. In European countries, the number of hospital admissions involving treatment for uterine fibroids ranges from 0.71 to 1.53 per 1000 women, and the estimated direct cost related to uterine fibroids ranges from two to five euros per woman [7].

Chinese medicine (CM) is an alternative and complementary medicine to treat gynecological diseases in Asian countries [8-10]. Among fibroid patients, three fourth of them have the experience of utilizing CM, which are considered to be with fewer side effects, less expensive [11,12], less subsequent surgery rate [13], and higher life quality than conventional western medicine [14]. Because of the high usage rate, the expense of CM in Taiwan has been covered by the government-run National Health Insurance (NHI) system since 1995 [15]. Despite of the above-mentioned advantages, it is still unclear that whether use of CM decrease the consumption of conventional western medicine, in the case of that CM users usually have more symptoms than CM nonusers [13], and are supposed to use more medical resources. Moreover, as CM generates a new component of medical cost, whether the use of CM decreases the total medical cost also needed to be clarified.

In comparing fibroid patients who were CM users with CM nonusers, we sought to determine: (1) if the use of CM correlates with the consumption of conventional western medicines that is specifically for symptoms related to fibroids, and, (2) if the use of CM changes the Nation’s health care cost for uterine fibroids.

## Methods

### Subjects

Patients with uterine fibroids were identified according to the International Classification of Diseases, 9^th^ Revision, Clinical Modification (ICD-9-CM) code 218 from 1996 to 2010 in the Longitudinal Health Insurance Database 2000 (LHID2000). The LHID2000 was set up by Taiwan’s National Health Research Institute and it contains chronological information about one million randomly selected individuals who were beneficiaries from 1996 to 2000. This study was approved by the Research Ethics Committee of the CMUH (CMU-REC-101-012).

### Study designs

The date of the first diagnosis was used as the entry date, and the endpoint date was December 31th 2010, the date of death, withdrawal from the insurance program, or underwent uterine surgery. The follow-up time was defined as the period from the diagnosis date to the endpoint date. CM users were defined as subjects who had received a mean orally administered CM treatment for more than fifteen days per year. The total fibroid - related consumption of western medicines was estimated by summarizing the number of days for which western medicines have been prescribed with a ICD-9-CM code of 218 from the diagnosis date to the endpoint date. Medical costs included insurance claim for the NHI reimbursement for outpatients (OPD), inpatients (IPD), emergency room (ER), and CM treatments that was related to uterine fibroid.

The examined variables included socio-demographic factors, including age (<20, 20 – 29, 30 -39, 40-49, and > 50 years), income level (<520, 520 -639, 640 – 759, and ≥ 759 USD, based on quartile), and occupation status (white collar, blue collar, and others), and endometriosis-related co-morbidities, including hypermenorrhea (ICD-9-CM 626.2), iron-deficiency anemia (ICD-9-CM 280), dysmenorrhea (ICD-9-CM 625.3), and infertility (ICD-9-CM 628 and 628.3).

### Statistical analysis

In terms of categorical and ordinal variables, CM users and CM nonusers were compared using *chi*-square tests and Wilcoxon rank sum test, respectively. Multivariate linear regression models were performed to identify the independent factors associated to the consumption of western medicines, and to assess annual costs in OPD, IPD, ER, and CM, as well as total annual medical cost. These adjusted models were controlled for age, occupation, income, and co-morbidities (hypermenorrhea, iron-deficiency anemia, dysmenorrhea, and infertility). Continuous data are expressed as mean ± SD and categorical data are expressed as number and percentage. All statistical analyses were performed using SAS software, version 9.1 (SAS Institute Inc., Carey, NC), and the significance level was set at a two-tailed *P* value of less than 0.05.

## Results

### Study subjects

A total of 44,122 women diagnosed with uterine fibrosis between 1996 and 2010 were extracted from the LHID2000 database with a mean follow-up time of 4.6 years. Among them, 11,412 patients were identified as CM users, and 32,710 patients were identified as CM nonusers. The mean age of CM users (41.4 years) was younger than CM nonusers (42.4 years) (Table 1). The proportions of patients among CM users who were white collar, and registered their insurance in Central Taiwan were higher than that among CM nonusers. Moreover, the proportions of patients who were co-morbid with iron-deficiency anemia, hypermenorrhea, dysmenorrhea, and infertility were all higher among CM users than they were among CM nonusers.

**Table 1:** **Comparison of socio-demographic factors and co-morbidities between Chinese medicine (CM) users and nonusers in patients with uterine fibroids**

	**CM**						
	**Nonusers**		**Users**		**Total**		***P*** **-value** ^**a**^
	**N = 32710**		**N = 11412**		**N = 44122**		
	**n**	**%**	**n**	**%**	**n**	**%**	
Age, years							< 0.0001
<20	202	0.6	47	0.4	249	0.5	
20-29	2634	8.1	1030	9.0	3664	8.3	
30-39	8998	27.5	3665	32.1	12663	28.7	
40-49	15559	47.6	5167	45.3	20726	47.0	
≥50	5317	16.5	1503	13.2	6820	15.5	
Mean ± SD	42.4 ± 8.9		41.4 ± 8.5		42.2 ± 8.8		
Income, US$ per month							0.1800
<520	8075	24.7	2725	23.9	10800	24.5	
520 - 639	8173	25.0	2966	26.0	11139	25.3	
640- 759	8556	26.2	2921	25.6	11477	26.0	
≥760	7906	24.2	2800	24.5	10706	24.3	
Mean ± SD	20098 ± 12630		20333 ± 12136		20158 ± 12505		
Occupational status							<0.0001
White collar	19243	58.8	6972	61.1	26215	59.4	
Blue collar	9900	30.1	3312	29.0	13212	29.9	
Others	3567	10.9	1128	9.9	4695	10.6	
Area							<0.0001
Northern Taiwan	16281	50.0	4820	42.2	21101	47.8	
Central Taiwan	5758	17.6	2656	23.3	8414	19.1	
Southern Taiwan	9265	28.3	3511	30.8	12776	29.0	
Eastern Taiwan and offshore islands	1406	4.3	425	3.7	1831	4.15	
Co-morbidity							
Iron-deficiency anemia	5259	16.1	2213	19.4	7472	16.9	<0.0001
Hypermenorrhea	8388	25.6	3650	32.0	12038	27.3	<0.0001
Dysmenorrhea	7456	22.8	4246	37.2	11702	26.5	<0.0001
Infertility	2570	7.9	1358	11.9	3928	8.9	<0.0001

^a^Chi-square test and Wilcoxon rank sum test.

### Factors associated with the consumption of conventional western medicine

The mean number of days of fibroid-related western medicine taking was 14.6 ± 50.6 days per year. After adjusting for socio-demographic factors (age, income, occupation, and area), and co-morbid covariates (hypermenorrhea, iron-deficiency anemia, dysmenorrhea, and infertility) in a multivariate linear regression model, CM users consumed less western medicine than CM nonusers (-10.5 days/year/patient, *P* < 0.0001). Patients who aged higher than 40 years, who were blue collar, who registered their insurance in Central Taiwan, and whose income were less than 520 USD/month tended to consume more conventional western medicine. Patients whose diseases were co-morbid with hypermenorrhea, iron-deficiency anemia (both *P* < 0.0001), and dysmenorrhea (*P =* 0.0202) also consumed more western medicine than who were not co-morbid with these illnesses. However, patients whose diseases were co-morbid with infertility tended to consumed less western medicine than whose diseases were not co-morbid with infertility (*P* = 0.0295) (Table 2).

**Table 2:** **Multivariate linear regression for number of days of fibroid-related treatment in patients with uterine fibroid**

	**Days/year** ^**a**^	**Estimate β**	**VIF** ^**b**^	**T**	***P***
CM					
Nonusers	17.2 ± 56.8	-	-	-	-
Users	7.4 ± 24.3	-10.49	1.03	-19.03	<0.0001
Age, years					
<20	3.5 ± 15.1	-	-	-	-
20-29	6.1 ± 27.3	4.22	14.49	1.29	0.1971
30-39	11.0 ± 41.3	8.75	37.28	2.73	0.0063
40-49	18.4 ± 58.3	15.41	45.19	4.82	<0.0001
≥50	15.0 ± 50.6	13.07	24.33	4.03	<0.0001
Income, US$ per month					
<520	14.7 ± 50.5	-	-	-	-
520 - 639	13.9 ± 48.7	-1.22	1.57	-1.78	0.0745
640- 759	17.1 ± 56.2	-0.36	1.94	-0.48	0.6300
≥760	12.8 ± 45.9	-2.46	1.67	-3.44	0.0006
Occupational status					
White collar	13.2 ± 47.3	-	-	-	-
Blue collar	17.2 ± 56.4	1.70	1.48	2.70	0.007
Others	15.1 ± 50.9	1.18	1.14	1.43	0.1514
Area					
Northern Taiwan	13.2 ± 47.7	-	-	-	-
Central Taiwan	17.0 ± 54.2	3.57	1.16	5.48	<0.0001
Southern Taiwan	16.0 ± 53.4	2.25	1.20	3.92	<0.0001
Eastern Taiwan and offshore islands	12.4 ± 44.4	-2.37	1.07	-1.92	0.0543
Hypermenorrhea					
No	13.1 ± 48.4	-	-		-
Yes	18.8 ± 55.8	3.99	1.08	7.21	<0.0001
Iron-deficiency anemia					
No	12.3 ± 45.8	-	-		-
Yes	26.2 ± 68.4	12.8	1.04	19.83	<0.0001
Dysmenorrhea					
No	14.8 ± 52.0	-	-		-
Yes	14.2 ± 46.3	1.32	1.13	2.32	0.0202
Infertility					
No	15.2 ± 51.7	-	-		-
Yes	9.5 ± 36.4	-1.88	1.08	-2.18	0.0295

^a^R-square = 0.0277 and adjusted R-square = 0.0274 in a multivariate linear regression model that was adjusted for age, income, occupation, area, and iron-deficiency anemia, infertility, excessive bleeding, and dysmenorrhea.

^b^VIF, variance inflation factor.

### Difference in the consumption of antianemics, hemostastics, analgesics, and hormone-related agents between CM users and nonusers

Taking all the subjects as a whole, multivariate linear regression revealed that CM users consumed less antianemics (-3.50 days/year/patient, *P* < 0.0001), hemostatics (- 1.89 days/year/patient, *P* < 0.0001), and hormone-related agents (-3.13 days/year/patient, *P* < 0.0001) than CM nonusers (Table 3). Stratification analysis showed that differences between CM users and nonusers in the consumption of antianemics substantially enlarged in patients with iron-deficiency anemia (-11.29 days/year/patient, *P* < 0.0001), that of hemostatics enlarged in patients with dysmenorrhea (-4.01 days/year/patient, *P* < 0.0001), and that of hormone-related agents enlarged in patients with hypermenorrhea (-7.84 days/year/patient, *P* < 0.0001). Moreover, the consumption of analgesics became significantly different between CM users and nonusers in fibroid patients with dysmenorrhea (-3.46 days/year/patient, *P* = 0.0013).

**Table 3:** **Consumption of antianemics, hemostatics, and analgesics in patients co-morbid with iron-deficiency anemia, hypermenorrhea, and dysmenorrhea**

	**CM nonusers**	**CM users**	**Linear regression model**	
	**days/year**	**days/year**	**β (SE)** ^**a**^	***P*** **-value**
**Total, N** ^**b**^	**32710**	**11412**		
Antianemics^c^	7.7 ± 39.3	4.9 ± 23.4	-3.50 (0.38)	< 0.0001
Hemostatics	4.1 ± 24.1	2.6 ± 12.2	-1.89 (0.24)	< 0.0001
Analgesics	38.0 ± 62.5	38.0 ± 50.3	-0.42 (0.64)	0.5145
hormone-related agents	16.4 ± 57.3	13.9 ± 39.7	-3.13 (0.58)	< 0.0001
**Iron-deficient anemia, n** ^**b**^	**5259**	**2213**		
Antianemics	30.9 ± 73.5	19.1 ± 43.4	-11.29 (1.68)	< 0.0001
Hemostatics	8.3 ± 34.6	5.2 ± 19.3	-3.36 (0.79)	< 0.0001
Analgesics	44.8 ± 70.2	42.8 ± 57.4	-2.24 (1.69)	0.1849
hormone-related agents	14.2 ± 40.6	14.0 ± 31.5	-1.20 (1.27)	0.34
**Hypermenorrhea, n**	**8388**	**3650**		
Antianemics	13.2 ± 49.6	8.5 ± 28.8	-5.17 (0.85)	< 0.0001
Hemostatics	7.9 ± 34.2	4.0 ± 13.5	-4.01 (0.59)	< 0.0001
Analgesics	41.7 ± 63.3	38.9 ± 47.4	-3.51 (1.16)	0.0026
hormone-related agents	24.0 ± 71.1	16.1 ± 39.0	-7.84 (1.26)	<0.0001
**Dysmenorrhea, n**	**7456**	**4246**		
Antianemics	7.9 ± 37.4	5.7 ± 24.1	-2.81 (0.62)	< 0.0001
Hemostatics	4.8 ± 24.8	2.8 ± 11.8	-2.13 (0.40)	< 0.0001
Analgesics	41.0 ± 61.8	37.2 ± 47.0	-3.46 (1.07)	0.0013
hormone-related agents	17.1 ± 56.2	12.5 ± 33.1	-4.96 (0.94)	<0.0001

^a^Multivariate linear regression model that was adjusted for age, income, occupation, area, and iron-deficiency anemia, infertility, excessive bleeding, and dysmenorrhea.

^b^N, subject number in a group; and n, subject number in a subgroup.

^c^Antianemics, including blood derivatives and iron; hemostatics, including coagulants and hemostatics; and analgesics, including non-steroid anti-inflammatory drugs and analgesics.

### Uterine fibroid-related medical cost between CM users and CM nonusers

The mean total medical cost of CM users was 503.9 USD, and that of nonusers was 6294 USD per patients. After adjusting for age, income, occupation, area, iron-deficiency anemia, infertility, hypermenorrhea, and dysmenorrhea, the total annual medical cost for treating CM users was 0.45-fold as much as that for treating CM nonusers (Table 4). Among the total medical cost, although the annual cost of Chinese medicine was 16.9 USD higher for CM users than that for CM nonusers, the annual cost of OPD (-13 USD/year, *P* < 0.0001), IPD (-5611 USD/year, *P* < 0.0001), and ER (-2.23 USD/year, *P* < 0.0001) for CM users were lower than that for CM nonusers.

**Table 4:** **Uterine fibroid-related cost in CM-users and nonusers**

	**Cost, US$/patient/year**		**Linear regression model** ^**a**^	
**Type** ^**b**^	**CM nonusers**	**CM users**	**β (SE)**	***P*** **value**
Total cost	6294 ± 41366	503.9 ± 4946	0.45 (1.05)	<0.0001
OPD	16.9 ± 146.5	4.57 ± 28.8	0.75 (1.02)	<0.0001
IPD	6274 ± 41317	480 ± 4833	0.26 (1.07)	<0.0001
surgery	2931 ± 19328	213 ± 1980	0.27 (1.07)	<0.0001
other (medication and admission)	3335 ± 25854	261.23 ± 3233	0.27 (1.07)	<0.0001
ER	2.111 ± 164.2	0.097 ± 3.51	0.60 (1.44)	<0.0001
CM	0.26 ± 7.37	18.7 ± 597.7	2.67 (1.17)	<0.0001

^a^Multivariate linear regression model for nature log transformation of cost and model was adjusted for age, income, occupation, area, iron-deficiency anemia, infertility, excessive bleeding, and dysmenorrhea.

^b^OPD, outpatients, IPD, inpatients, ER, emergency room, and CM, Chinese medicine.

## Discussion

CM is used by patients with uterine fibroids as an alternative to conventional western medicine. This study compared the consumption of conventional western medicine and the exact medical cost between CM users and nonusers in fibroid patients. Although more CM users were co-morbid with iron-deficiency anemia, hypermenorrhea, dysmenorrhea, and infertility, they consumed less fibroid-related western medicines than CM nonusers. The difference in western medicine consumption between the two groups enlarged in patients with co-morbidities. Moreover, the utilization of CM did generate an extra medical cost, however, the total NHI-reimbursed annual health care fee per patient in CM users was substantially lower than that in CM nonusers.

Social-demographic trends regarding CM users in this study, which were extracted from a population-based database from 1996 to 2010, found that CM users in fibroid patients were more likely to be younger and white collar than nonusers. These trends was coincident with a previous study that extract patients from 2000 to 2003 [13], and also with studies on female-specific diseases, including gynecological and breast cancer [16,17] and endometriosis [18]. In terms of co-morbidities, including iron-deficiency anemia, hypermenorrhea, dysmenorrhea, and infertility, this study showed that fibroid patients with co-morbidities tended to use CM, implying severe patients with symptoms were more likely to seek for alternative treatments. Several studies have also reported that most alternative medicine users are with symptoms of chronic diseases and poor health status [19-21], and “suffering from disease” is considered one of an important factors that triggers patients to use CM [19].

Similar to those being associated to CM, high frequencies of taking conventional western medicine were associated with co-morbidities of hypermenorrhea, iron-deficiency anemia, and dysmenorrhea were associated with. However, patients who were co-morbid with infertility consumed less conventional western medicine than patients who were not co-morbid with infertility. Other social-demographic factors that associated with high consumption of conventional western medicine included age higher than 40, being blue collar, income less than 520 USD/month, and registration of the insurance in Central and Southern Taiwan. In terms of using CM, CM users were less likely to consume conventional medicine, indicating that CM users reduced the use of conventional western medicine when they chose CM to treat diseases.

Antianemics, hemostatics, analgesics, and hormone-related agents are commonly suggested medicines to control fibroid-related symptoms, i.e., iron-deficient anemia, hypermenorrhea, and dysmenorrhea [4]. As medications are only used in patients with well-defined signs or symptoms, among patients with a specific co-morbidity, the difference in the consumption of a co-morbidity-specific medicine implied that CM substituted traditional western medicine to treat this co-morbidity for CM users. For example, among patients co-morbid with hypermenorrhea, the low consumption of hemostatics in CM users implied CM substituted hemostatics to control symptoms of hypermenorrhea. Our data implied that CM might be useful to control symptoms caused by uterine fibroid. Animal studies and clinical studies have reported that CM might have two beneficial effects on uterine fibroid. First, CM is able to treat the symptoms related to uterine fibroid. Second, CM is able to reduce the size of fibroids. An animal study has shown that Shao-fu-zhu-yu decoction, a commonly used CM formula for dysmenorrhea, has been shown to relieve uterine contraction pain by its peripheral analgesic activities [22]. Another animal study reported that Bak-foong pills are able to directly down-regulate mouse uterine tone [23]. However, clinical trials regarding the efficacy of CM on uterine fibroids are not of good quality. A systemic review reported that only two of 21 clinical trials are with strict randomization and blinding processes. These two rigorous randomized controlled trials reported that herbal preparation made fibroids to shrink, but the size reduction of fibroids by herbal preparation did not differ from the efficacy of mifepristone [24]. The present study provided epidemiological evidence showing difference in consumption of conventional western medicine between CM users and nonusers among fibroid patients, implying therapeutic effects to release symptoms caused by fibroids.

Complementary and alternative medicine is considered less expensive than conventional western medicine [11,12], and the mean cost per visit is lower for CM than for conventional western medicine [9]. However, since the use of CM generates a component of medical cost, whether using CM decreases the total medical cost is controversial. A review article concludes that there is still a lack of cost-effectiveness research in CM [25]. This study found that the use of CM did reduce total medical cost paid by nation’s health-care system for uterine fibroid. Although there was an extra expenditure in CM (18.7 USD/year/patient) spent by CM users, this extra expenditure was much less than the expenditure in conventional western medicine in both of the groups. Moreover, as CM users reduced the use of western medicine, the total medical reimbursement from NHI for CM users was 5610 UDS/year/patient less in CM users than in nonusers. The costs of uterine fibroids for OPD, IPD, and ER in CM users were all lower than that in CM nonusers. The major difference in medical cost between CM users and nonusers was the in-patient cost. CM users saved more than 2,622 dollars in the cost of surgery and 2,987 dollars in the cost of hospital admission and inpatient medication per patient annually, indicating CM users underwent less surgery and less hospitalization than CM nonusers. The present group has reported that CM users were more unlikely to under uterine surgery, including hysterectomy and myomectomy, with a hazard ratio of 0.18 [13]. This study confirmed that the decreased surgery rate in CM users reduced health care spending in uterine fibroid.

Using a database from the NHI, which is a government-run, single-payer national health insurance program that insures over 97% of citizens and over 99% of health-care institutes [26,27], this study provided epidemiological evidence showing different amount of conventional western medicine consumption and different medical cost between CM users and nonusers among patients with uterine fibroids. However, the first limitation of this study was that we could not verify whether patients underwent other forms of alternative medicines that are not covered by NHI, although the proportional of patients who took those alternative medicines was presumed small because of their high prices. The second limitation of this retrospective study was that it could not directly provide evidence for the efficacy of CM, but could only find a correlation between using CM and low consumption of western medicine and low cost. This correlation could also possibly come from the difference in healthcare seeking behavior between CM users and nonusers. Nevertheless, whatever the underlying reason was, this study highlighted that CM was associated with low consumption of conventional western medicine and low medical cost in fibroid patients. For practitioners, this study provided evidence that CM might be a potential therapeutic substitute for conventional western medicines to treat several symptoms of uterine fibroids. Moreover, for insurance policy makers, this study suggested that CM might be a proper area for health insurance to cover in fibroid patients.

## Conclusion

This study revealed that CM reduced the consumption of conventional medicine, including total fibroid-related conventional medicine and three categories of conventional medicines (antianemics, hemostatics, and hormone-related agents) that are commonly used to release symptoms of fibroids. Moreover, although using CM increased a small amount of medical cost, the total medical cost for treating fibroid was less in CM users than in nonusers. Our results implied that CM might be a potential therapeutic substitute for conventional medicines to treat uterine fibroids with low cost.

## Abbreviations

CM: Chinese medicine
IPD: Inpatient department
ER: Emergency room
ICD-9-CM: International Classification of Diseases, 9^th^ Revision, Clinical Modification
LHID2000: Longitudinal Health Insurance Database 2000
NHI: National Health Insurance
NSAID: Non-steroid anti-inflammatory drugs
OPD: Outpatient department
USD: US dollar
